# Glucocorticoids in preterm human milk

**DOI:** 10.3389/fnut.2022.965654

**Published:** 2022-09-27

**Authors:** Mariana Muelbert, Tanith Alexander, Mark H. Vickers, Jane E. Harding, Laura Galante, Frank H. Bloomfield, the DIAMOND study group, Mariana Muelbert

**Affiliations:** ^1^Liggins Institute, University of Auckland, Auckland, New Zealand; ^2^Neonatal Unit, Kidz First, Middlemore Hospital, Auckland, New Zealand; ^3^Newborn Services, Auckland City Hospital, Auckland, New Zealand; ^4^ Department of Paediatrics: Child and Youth Health.; ^5^Department of Newborn Services, Mater Mothers’ Hospital, Brisbane, QLD, Australia; ^6^Mater Research Institute, The University of Queensland, Brisbane, QLD, Australia; ^7^Department of Statistics, Faculty of Science, University of Auckland, Auckland, New Zealand; ^8^Food and Bio-based Products, AgResearch Grasslands, Palmerston North, New Zealand; ^9^Department of Nutrition, Faculty of Medical and Health Sciences, University of Auckland, Auckland, New Zealand; ^1^Liggins Institute, University of Auckland, Auckland, New Zealand; ^2^Neonatal Unit, Kidz First, Middlemore Hospital, Auckland, New Zealand

**Keywords:** cortisol, cortisone, breastmilk, lactation, nutrition, antenatal corticosteroids, moderate preterm, late preterm

## Abstract

**Background:**

Glucocorticoids (GCs), cortisol and cortisone, are essential regulators of many physiological responses, including immunity, stress and mammary gland function. GCs are present in human milk (HM), but whether maternal and infant factors are associated with HM GC concentration following preterm birth is unclear.

**Materials and methods:**

HM samples were collected on postnatal day 5 and 10 and at 4 months’ corrected age (4m CA) in a cohort of moderate- and late-preterm infants. GCs in HM were measured by liquid chromatography-tandem mass spectrometry. Relationships between GCs in HM and both maternal and infant characteristics were investigated using Spearman’s correlations and linear mixed models.

**Results:**

170 mothers of 191 infants provided 354 HM samples. Cortisol concentrations in HM increased from postnatal day 5–4m CA (mean difference [MD] 0.6 ± 0.1 ng/ml, *p* < 0.001). Cortisone concentration did not change across lactation but was higher than cortisol throughout. Compared to no antenatal corticosteroid (ANS), a complete course of ANS was associated with lower GC concentrations in HM through to 4m CA (cortisol: MD –0.3 ± 0.1 ng/ml, *p* < 0.01; cortisone MD –1.8 ± 0.4 ng/ml, *p* < 0.001). At 4m CA, higher maternal perceived stress was negatively associated with GC concentrations in HM (cortisol adjusted beta-coefficient [aβ] –0.01 ± 0.01 ng/ml, *p* = 0.05; and cortisone aβ –0.1 ± 0.03 ng/ml, *p* = 0.01), whereas higher postpartum depression and maternal obesity were associated with lower cortisone concentrations (aβ –0.1 ± 0.04 ng/ml *p* < 0.05; MD [healthy *versus* obese] –0.1 ± 0.04 ng/ml *p* < 0.05, respectively). There was a weak positive correlation between GC concentrations in HM and gestational age at birth (*r* = 0.1, *p* < 0.05). Infant birth head circumference *z*-score was negatively associated with cortisol concentrations (aβ –0.01 ± 0.04 ng/ml, *p* < 0.05). At hospital discharge, fat-free mass showed a weak positive correlation with cortisol concentrations (*r* = 0.2, *p* = 0.03), while fat mass showed a weak negative correlation with cortisone concentrations (*r* = –0.25, *p* < 0.001).

**Conclusion:**

The mammary gland appears to protect the infant from cortisol through inactivation into cortisone. Maternal and infant characteristics were associated with concentration of GCs in HM, including ANS, stress and depression scores, obesity, gestational age and infant size. The effects of HM glucocorticoids on long-term health outcomes requires further research.

## Introduction

Human milk (HM) can act as a messenger between mother and the infant, conveying nutritional, non-nutritional, immune, and biologically active compounds involved in various signaling pathways key for development (1–3). Hormones in HM confer individualized cues about maternal health and environment that may influence infant metabolism, growth and postnatal development with potential long-term effects (3–5).

The hypothalamic-pituitary-adrenal (HPA) axis is essential for orchestrating response to stressors and the circadian cycle (6). During pregnancy, the maternal HPA axis undergoes dramatic changes, with cortisol concentrations progressively rising to a peak in the third trimester, partially in response to increasing secretion of placental corticotrophin-releasing hormone (CRH) (6, 7). To protect the fetus from excessive exposure to cortisol, the placenta expresses 11β-hydroxysteroid dehydrogenase type 2 (11β-HSD2), an enzyme that converts cortisol into its inactive form, cortisone (7, 8). By late gestation (around 34 weeks), fetal adrenal production of the glucocorticoids (GCs) cortisol and cortisone gradually increases and is essential for maturation of fetal organs including lungs, thyroid and the gastrointestinal tract, in preparation for the postnatal environment (8, 9). For mothers at risk of preterm birth, administration of antenatal glucocorticoids accelerates fetal lung maturation and reduces neonatal morbidity and mortality (10). Following birth, circulating maternal cortisol concentrations and HPA axis activity gradually return to pre-pregnancy levels (7, 11).

Data from animal and human research suggest that exposure to endogenous (i.e., hypertension, insulin resistance, undernutrition, obesity) and exogenous (i.e., depression, social deprivation) maternal stressors during pregnancy and the perinatal period is associated with a higher risk for obesity and metabolic dysfunction in the offspring (6, 9). Maternal stress/anxiety, and inflammation or infection during pregnancy can result in excessive maternal cortisol concentrations and, although 11β-HSD2 is capable of metabolizing up to 90% of maternal cortisol, there may be increased fetal exposure to GCs during the critical period of HPA axis development (7, 8). Considering the central role of the HPA axis in key metabolic pathways, insults during HPA axis development are proposed as a mechanism by which adverse perinatal exposures may influence life-long health (6, 9).

Together with prolactin and insulin, maternal GCs also play a central role in lactogenesis, stimulating mammary gland differentiation and structural changes to support HM synthesis and secretion (12). The GCs cortisol and cortisone are two of many hormones present in HM (3–5). Concentrations of cortisol and cortisone in HM follow the maternal HPA axis activity (13–15), displaying the same circadian rhythm, peaking in the morning and slowly decreasing throughout the day (13, 14, 16). A diurnal rhythm of GCs is present in term and preterm infants from 1 month postnatal and corrected age, respectively (17, 18). The establishment of circadian rhythm is essential to control normal sleep–wake cycles, respiratory rate, body temperature, digestion, metabolism, hormone release, and other important physiological functions (5, 6). However, compared to full-term infants, those infant born extremely preterm (<28 weeks) have blunted cortisol reactivity to acute stress at 4 months chronological age (19) and flattened diurnal cortisol slope across the first year of life (20), possibly indicating suppression of HPA axis activity.

Whether GCs in HM relate to maternal and infant health remains unclear, with studies reporting conflicting associations between GCs in HM and the effects of lactation stage (21–23), gestational age (13, 21, 24, 25), maternal stress (16, 24, 26), infant adiposity (27, 28) and temperament (23, 29). Moreover, only small studies have investigated the concentration of GCs in preterm HM and mainly after very preterm birth (<32 weeks’ gestation) (13, 21, 25). Therefore, the aim of the present study was to determine HM concentrations of cortisol and cortisone from mothers of moderate to late preterm infants and explore potential associations with maternal and infant characteristics.

## Materials and methods

### Study population

We undertook a prospective cohort analysis nested within the DIAMOND trial (ACTRN12616001199404; Health and Disability Ethics Committee 16/NTA/90) (30). Briefly, this factorial randomized controlled trial compared three nutritional interventions in moderate and late preterm infants admitted to four neonatal nurseries in New Zealand. The interventions were: (i) dextrose solution *versus* amino acid solution (with or without lipid emulsion at the clinician’s discretion) as the intravenous fluid; (ii) breastmilk as the only milk *versus* a breastmilk substitute if maternal breastmilk supply did not meet infant requirements, and (iii) exposure of the infant to smell and taste of milk prior to each feed by gastric tube, *versus* no exposure. Eligibility criteria were: birth between 32^+0^ and 35^+6^ weeks’ gestation; intravenous lines *in situ*; mother intending to breastfeed, and no congenital abnormality that might impact upon growth and development.

Maternal age, ethnicity, education, postcode, and clinical information (maternal diabetes, antenatal steroid administration, delivery mode), and infant characteristics (sex, gestational age at birth, anthropometric measures) were collected prospectively. In New Zealand, postcode of domicile is used to generate a social deprivation index (the New Zealand Deprivation [NZdep] Index) in the Classification Coding System from Statistics New Zealand (31) with a decile scale from 1 to 10, representing low to high social deprivation. Ethnicity was self-identified and prioritized according to New Zealand’s Ministry of Health protocols (32). For analysis, ethnicity was grouped into Caucasian/European; Asian (Asia, South-East Asia, and Indian subcontinent); Pasifika (South-Western Pacific); Māori (New Zealand Māori); and Other. Antenatal corticosteroid (ANS) administration was classified as a complete course (>1 dose given, with the first dose >24 h before birth), incomplete (1 dose given <24 h before birth), or no ANS received.

Two self-completed questionnaires were used to assess symptoms of depression (Edinburgh Postnatal Depression Scale, EPDS) and stress (Perceived Stress Scale, PSS) around postnatal day 10 and when the infant was 4 months’ corrected age (4m CA, ± 2 weeks, defined from 40 weeks’ gestation). The EPDS asked how mothers felt during the previous week prior to completion of the questionnaire. The maximum score is 30 and mothers who scored 10 or above possibly experienced symptoms of postnatal depression (33, 34). The PSS questionnaire asked how mothers felt during the previous month using a 14-item instrument. Scores ranging from 0 to 13, 14 to 26, and 25 to 40 points were considered low, moderate and high perceived stress, respectively (35).

### Nutrition and growth

Data about nutritional intake and growth during hospital stay were collected prospectively. Weight, length and head circumference were recorded weekly until hospital discharge and again at 4m CA. *Z*-scores were calculated based on the Fenton preterm growth charts for in-hospital growth (36) and on the World Health Organization (37) growth chart at 4m CA. Linear growth was calculated based on changes in *z*-score between two time points of interest. Growth velocity from birth to discharge was calculated using the exponential method (38). Body composition was measured by air displacement plethysmography (PEA POD^^®^^, COSMED, Concord, CA, USA) in a sub-set of infants at hospital discharge and at 4m CA.

### Sample collection

HM samples were collected during the morning on postnatal day 5 (±2 days) and 10 (±2 days) and at 4m CA (±2 weeks) if mother was still breastfeeding. Mothers were requested to express milk from their right breast using an electronic breast-pump (Medela Symphony^®^, Switzerland) into disposable sterile bottles (Medela^®^, Switzerland) at least 2–3 h after the previous milk expression. After the right breast was completely emptied, the total volume of expressed HM was vortexed for 2 min at high speed to ensure homogeneity and 2 ml was collected using a sterile enteral syringe (BD, Singapore). The collected HM was aliquoted into low-protein-binding microtubes (Eppendorf, Germany) and stored at –80°C until analysis. The lactation stage was defined as colostrum (samples collected around postnatal day 5), transitional HM (samples collected around postnatal day 10), and mature HM (samples collected at 4m CA). The cohort reported here includes mothers who provided at least one HM sample during the study period and their infants.

### Analysis of glucocorticoids in preterm human milk

HM GCs were measured by liquid chromatography-tandem mass spectrometry, as described previously (15). Briefly, 200 μl of HM heated at 37°C for 10 min was vortexed for 20 s before adding sample to glass tubes with internal standards (12 ng/ml cortisol D4 and 60 ng/ml corticosterone D8, prepared in milli-Q water). Steroids were then extracted using 1 ml ethyl acetate (Merck, Germany); the top organic layer was removed and vacuum dried (Savant, SC250EX, Thermo Scientific, USA) for 1 h, and then reconstituted with 60 μl 50% methanol (Merck, Germany) in water and transferred to Ultra High Pressure Liquid Chromatography (UPLC) injector vials. The UPLC Mass Spectrometer (MS) used a Vanquish pump and auto-sampler followed by an Ion Max APCI source on a Quantiva triple-quadrupole mass spectrometer, controlled by Xcalibur software (Thermo Electron Corporation, San Jose, CA, USA). The chromatography was performed using Phenomenex C18 F5 column (100 × 2.1 mm, 2.6 μm particle size) at 40°C.

GC concentrations were calculated from a standard curve generated for each GC relative to its internal standard, cortisol 0.05–100 ng/ml and cortisone 0.025–50 ng/ml, diluted into charcoal stripped human plasma (SeraCon II CD, Seracare, Milford MA, USA) and extracted in the same way as the samples for each assay. The limits of quantification for cortisol and cortisone were 0.05 and 0.025 ng/ml, respectively. The limits of detection for cortisol and cortisone were 0.03 and 0.01 ng/ml, respectively.

### Statistical analyses

Concentration of cortisol and cortisone are presented in ng/ml of HM. Concentrations of GCs were assessed for normality using Shapiro–Wilk test. Logarithmic transformation did not result in a normal distribution, and therefore descriptive analyses were performed with and without logarithmic transformation. As similar results were obtained, further statistical analyses were performed without logarithmic transformation. Spearman correlation was used to assess the relationship between GC concentrations and maternal and infant characteristics, and these were then further explored using linear mixed models including maternal study number as a random effect and lactation stage as a fixed effect and adjusted for ANS course. Models exploring infant factors were further adjusted for infant sex. Longitudinal changes in GC concentrations were explored using linear mixed models including maternal study number (within-subjects effect) and ANS course (fixed effect). Missing datapoints were estimated using restricted maximum likelihood. Statistical analyses were performed in R v4.1.0 software (R Core Team, Vienna, Austria) using the base “*Stats*” library (v3.6.1). Linear mixed models were conducted using R library “*lme4*” (v 1.1-26) and *post hoc* tests were conducted using R library “*emmeans*” (v1.5.5). A *p* value of ≤ 0.05 was considered significant, with false discovery rate-adjusted *p* value (FDR, Tukey Honest Significant Difference) for multiple comparisons. Data are presented as frequency (%), mean (standard deviation, SD) or median (range, minimum–maximum).

## Results

### Study population

**FIGURE 1:**
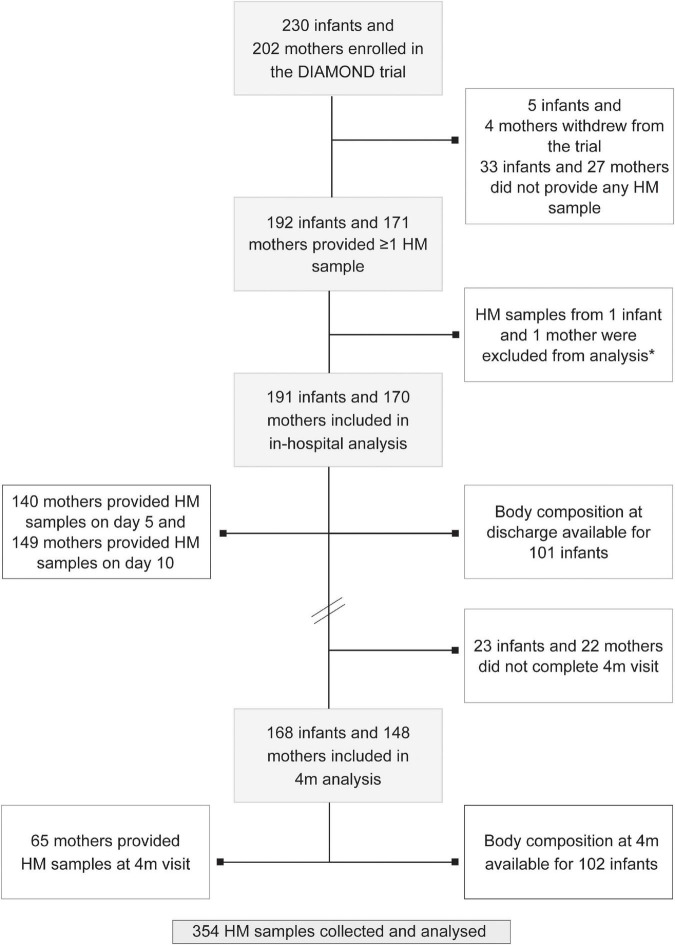
Study flowchart. Note that numbers do not add up as some mothers provided >1 sample and some mothers gave birth to multiples (twins and triplets). HM: human milk; 4m: 4 months follow-up visit. *Outlier sample excluded from analysis.

In total, 230 babies were enrolled in the DIAMOND trial between March 2017 and August 2019. Of these, 171 mothers of 192 infants provided samples HM samples. One mother-infant pair was excluded from analysis due to extremely high HM cortisol and cortisone concentrations (23 and 20.5 ng/ml, respectively), leaving 170 mothers of 191 babies who provided 354 HM samples (day 5: *n* = 149, day 10: *n* = 140 and 4m CA: *n* = 65) included in this analysis (Figure 1). Mean (standard deviation, SD) postnatal age at HM sample collection was 5 (1), 9 (1) and 160 days, and 70% of samples were collected before midday. Most mothers received at least some ANS (79%), had at least post-secondary education (70%) and were of Caucasian or Asian ethnicity, with Māori ethnicity under-represented compared with national birthing data (12 vs 25% nationally; Table 1). Mothers residing in areas of high social deprivation (NZDep quintiles 4 and 5) were over-represented (47%) and 60% were overweight or obese. Infants were born at a median (range) gestational age of 33 weeks, 12% were small-for-gestational age (SGA), 26% were multiples and 57% were boys (Table 1). Average (SD) length of hospital stay was 22 days and 78% of mothers were providing exclusively HM to their babies at hospital discharge (breastfeeding and/or expressed HM). However, only 19% of mothers were exclusively breastfeeding at 4m CA (Table 2).

**TABLE 1:** Study population characteristics.

Maternal characteristics (*n* = 170)	
Age, years (mean, SD)	31 (6)
**Ethnicity**	
Caucasian	66 (39)
Māori	20 (12)
Asian	54 (32)
Pasifika	27 (15)
Other	3 (2)
**New Zealand Deprivation Index**	
Q1 (1,2)	27 (16)
Q2 (3,4)	39 (23)
Q3 (5,6)	24 (14)
Q4 (7,8)	30 (18)
Q5 (9,10)	40 (29)
**Education level**	
Secondary education or lower	51 (30)
Post-secondary education	30 (18)
Tertiary education (university degree or higher)	89 (52)
**Number of samples at each time point**	
Day 5	149 (42)
Day 10	140 (39)
4 months	65 (18)
**Pregnancy characteristics (*n* = 170)**	
**Antenatal steroid course**	
None	35 (21)
Incomplete (first dose <24 h from birth)	38 (22)
Complete	97 (57)
Maternal diabetes	35 (21)
Caesarean section	101 (59)
**Maternal BMI at 4 months (*n* = 134)**	
Healthy (BMI ≤24.9 Kg/m^2^)	53 (40)
Overweight (BMI 25-29.9 Kg/m^2^)	32 (23)
Obese (BMI ≥30 Kg/m^2^)	49 (37)
**Infant characteristics (*n* = 191)**	
Boys	109 (57%)
Gestational age, weeks (median, IQR)	33 (32–35)
Moderate preterm (32^+0^ to 33^+6^ weeks’ gestation)	100 (52%)
**Size at birth**	
SGA	24 (12)
AGA	160 (84)
LGA	7 (4)
Twins/Triplets	50 (26)
Duration of hospital stay, days	22 (11)

Data are *n* (%) unless otherwise stated. Q, quintile of social deprivation; BMI, body mass index; IQR, interquartile range; SGA, small-for-gestational age; AGA, appropriate-for-gestational age; LGA, large-for-gestational age.

**TABLE 2:** Feeding practices and infant growth.

Feeding practices *n* (%)		
**At discharge (*n* = 168)**		
Exclusively HM	133 (78)	
Mainly HM (≥50% HM)	18 (11)	
Mainly IF (<50% HM)	12 (7)	
Exclusively IF	5 (3)	
At 4 months (*n* = 148)		
Exclusively HM	28 (19)	
Partially breastfeeding (HM + food, no IF)	20 (13)	
Mixed feeding (HM + IF + foods)	37 (25)	
IF feeding	63 (43)	
**Anthropometric measures**		
At birth (*n* = 191)		***z*-score**
Weight, g	2,102 (420)	–0.1 (0.9)
Length, cm	44.5 (3.0)	0.2 (1.1)
Head circumference, cm	31.2 (1.6)	0.3 (0.9)
**At hospital discharge (*n* = 189)**		
Weight, g	2,515 (330)	–0.8 (0.8)
Length, cm	47.5 (2)	–0.1 (0.9)
Head circumference, cm	33.0 (1.2)	–0.03 (0.7)
**At 4m CA (*n* = 168)**		
Weight, g	6,580 (867)	–0.1 (1)
Length, cm	63.7 (2.5)	0.5 (1.1)
Head circumference, cm	41.6 (1.3)	0.6 (0.9)
**Growth birth to discharge, median (min, max)**		
Growth velocity, g/Kg/day	7.7 (–19.3, 19)	
Weight *z*-score change	–0.7 (–1.8, 0.5)	
Length *z*-score change	–0.4 (–2.5, 2.3)	
Head circumference *z*-score change	–0.4 (–2.2, 1.4)	
**Growth birth to 4m CA, median (min, max)**		
Weight *z*-score change	–0.03 (–2.8, 2.1)	
Length *z*-score change	0.3 (–2.8, 4.8)	
Head circumference *z*-score change	0.3 (–2.1, 2.4)	
**Body composition at discharge (*n* = 101)**		**Kg**
Fat mass, %	10 (4)	0.25 (0.1)
Fat-free mass, %	90 (4)	2.2 (0.2)
Fat mass index, Kg/m^2^	1.1 (0.6)	
Fat free mass index, Kg/m^2^	9.8 (0.6)	
**Body composition at 4m CA (*n* = 102)**		**Kg**
Fat mass, %	25.3 (5)	1.6 (0.4)
Fat-free mass, %	74.7 (5)	4.8 (0.5)
Fat mass index, Kg/m^2^	4.1 (1.0)	
Fat free mass index, Kg/m^2^	11.8 (0.9)	

Data presented as *n* (%) or mean (SD), unless stated otherwise. HM, human milk; IF, infant formula; 4m CA, 4 months corrected age.

### Lactation stage

**FIGURE 2:**
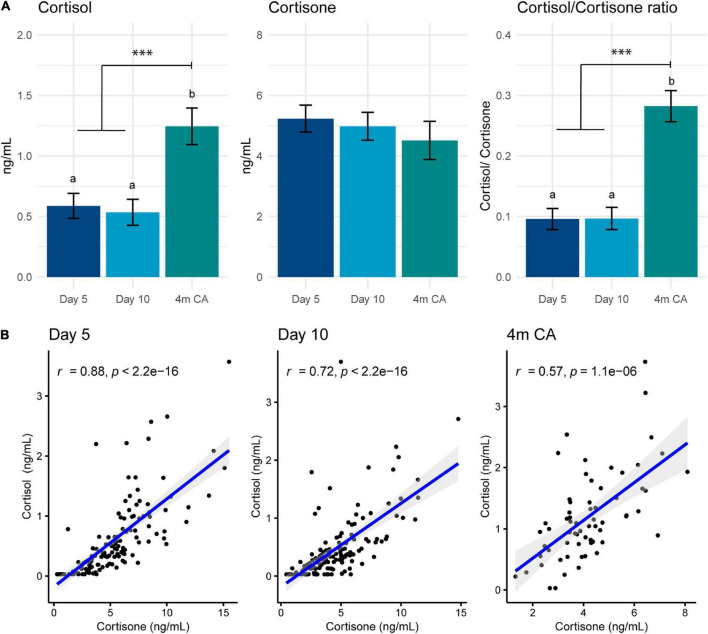
**(A)** Human milk concentrations of cortisol, cortisone and the cortisol-to-cortisone ratio at different lactation stages. Data are mean and standard error. Statistically significant differences are represented by different letters above the bars. ^***^p < 0.001 **(B)** Correlations between cortisol and cortisone at different lactation stages: postnatal day 5 (left), day 10 (middle), and 4 months’ corrected age (right). Blue line represents Spearman correlation coefficient and shaded area the 95% confidence interval. 4m CA: 4 months’ corrected age.

The median concentration of cortisol in preterm HM was 0.5 (range 0.03–3.7) ng/ml and of cortisone was 4.3 (0.3–15.5) ng/ml, with cortisone concentrations higher than cortisol concentrations throughout. Cortisol concentration was significantly higher in mature HM than in samples collected on days 5 and 10 (mean (standard error [SE]) *day 5*: 0.6 (0.05) ng/ml; *day 10*: 0.5 (0.05) ng/ml; *4m CA*: 1.2 (0.1) ng/ml, *F*_(2, 248)_ = 35.7, *p* < 0.001; Figure 2A). In contrast, cortisone concentrations did not change significantly over time (*day 5*: 5.2 (0.2) ng/ml; *day 10*: 5.0 (0.2) ng/ml; *4m CA*: 4.5 (0.3) ng/ml, *F*_(2, 234)_ = 2.2, *p* = 0.1). The ratio between cortisol and cortisone increased from early to late lactation (*p* < 0.001; Figure 2A). Concentrations of cortisol and cortisone were highly correlated throughout lactation; however, the strength of the correlation decreased as lactation progressed (Figure 2B).

Total volume of HM expressed at time of sample collection on days 5 and 10, but not 4m CA, showed a weak negative correlation with cortisol concentrations (*day 5*: *r* = –0.2, *p* = 0.01; *day 10*: *r* = –0.2, *p* = 0.03; *4m CA*: *r* = –0.03, *p* = 0.7) and cortisol-to-cortisone ratio (*day 5*: *r* = –0.2, *p* < 0.001; *day 10*: *r* = –0.2, *p* < 0.01; *4m CA*: *r* = 0.1, *p* = 0.4). Cortisone concentration was not correlated with total volume of HM expressed (*day 5*: *r* = –0.1, *p* = 0.4; *day 10*: *r* = 0.04, *p* = 0.6; *4m CA*: *r* = –0.1, *p* = 0.6).

### Perinatal factors

**FIGURE 3:**
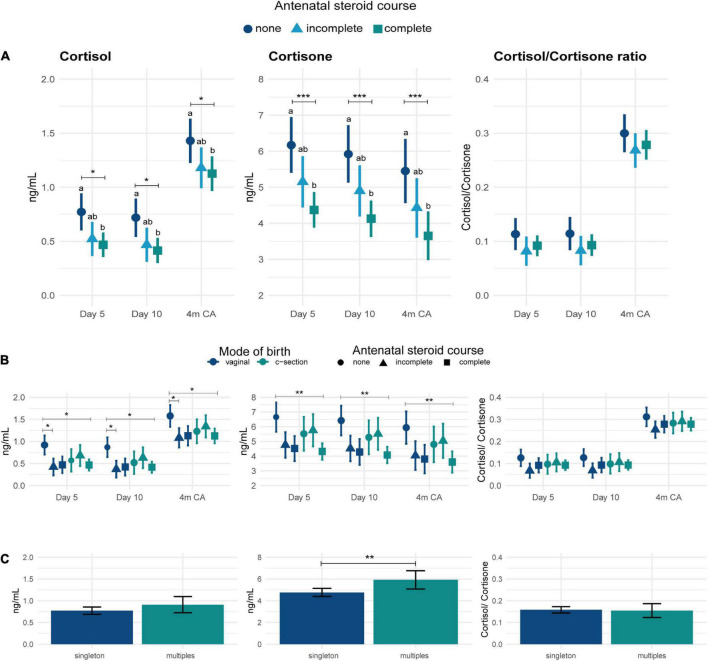
Concentrations of cortisol, cortisone and cortisol-to-cortisone ratio in human milk. **(A)** After different antenatal steroid courses and times of sample collection, **(B)** after different modes of birth (dark blue: vaginal birth; green: c-section) and antenatal steroid courses (circle: none; triangle: incomplete; square: complete) and time of sample collection, and **(C)** after singleton or multiple pregnancy. Data are mean concentration (ng/ml) and standard error. Statistically significant differences are represented by different letters above the symbols. *p < 0.05; **p < 0.01; ***p < 0.001. 4m CA: 4 months corrected age.

Mothers who received a complete course of ANS had significantly lower concentrations of cortisol (mean difference [MD] –0.3 (0.1) ng/ml, *p* < 0.01) and cortisone (MD –1.8 (0.4) ng/ml, *p* < 0.001) in HM than mothers with no ANS, regardless of length of gestation (moderate *versus* late preterm birth), and this association persisted to 4m CA (Figure 3A).

Overall, there was no significant association between GC concentration in HM and mode of birth (*p* = 0.7; Supplementary Table 1); however, mothers who birthed vaginally and received ANS had lower HM cortisol concentrations than mothers who birthed vaginally and received no ANS through to 4m CA (MD –0.5 (0.1) ng/ml, *p* = 0.04; mode of birth * ANS interaction *F*_(2,155)_ = 3.9, *p* = 0.02; Figure 3B). Although the pattern was similar for cortisone between mothers who birthed vaginally and received ANS and those who birthed vaginally and received no ANS, this was not statistically significant (MD –2.1 (0.6) ng/ml, *p* = 0.09; birth mode * ANS interaction *F*_(2,160)_ = 2.3, *p* = 0.1). Among women who birthed by Caesarean section, there were no differences in cortisol or cortisone concentrations between those who had and had not received ANS (Figure 3B).

Compared to mothers of singletons, HM from mothers of twins or triplets had higher cortisone concentrations (MD = 1.1 (0.4) ng/ml; *F*_(1,164)_ = 7.0, *p* = 0.01, Figure 3C). The concentration of GCs in preterm HM were not associated with maternal age, ethnicity, socioeconomic deprivation, level of education or maternal diabetes (Supplementary Table 1).

### Maternal stress and postnatal depression

**FIGURE 4:**
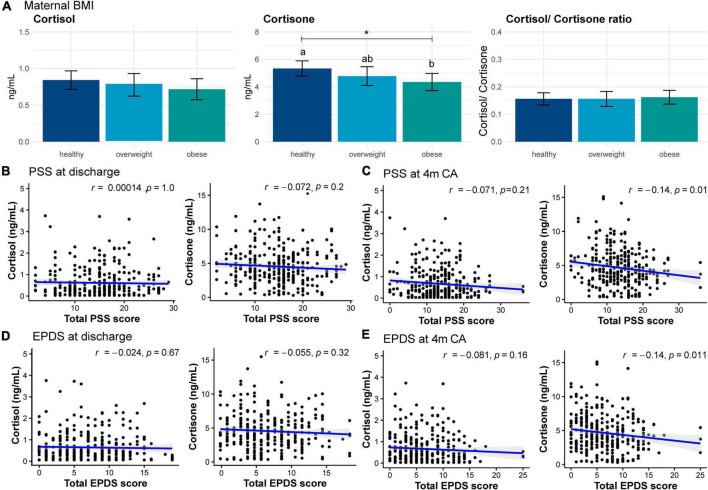
Cortisol and cortisone concentrations in human milk in women with different. **(A)** Body mass index (BMI), **(B)** Total Perceived Stress Score (PSS) at discharge, **(C)** Total PSS at 4 months, **(D)** Total Edinburgh Postnatal Depression Scale (EPDS) at discharge, and **(E)** Total EPDS at 4 months (4 m). Blue line and shaded area indicate regression line and 95% confidence interval, respectively. Data are mean concentration (ng/ml) and standard error. Statistically significant differences are represented by different letters above the bars. *p < 0.05.

Median maternal PSS score was 15 (2–29) at day 10 and 13 (0–36) at 4m CA, with 5% of mothers having scores indicative of high level of stress at day 10 and 3% at 4m CA (Supplementary Table 1). GC concentration in HM was not different amongst mothers with different perceived levels of stress at either time point (Supplementary Table 1). PSS total scores at hospital discharge was not correlated with GC concentration in HM (Figure 4B). PSS total score at 4m CA exhibited a weak negative correlation with GC concentration in HM, which was statistically significant for cortisone (*r* = –0.1, *p* = 0.01) but not cortisol (*r* = –0.1, *p* = 0.2; Figure 4C). In the adjusted model, total PSS score at 4 months was negatively associated with HM concentrations of both cortisol (adjusted beta coefficient [aβ] –0.01 (0.01) ng/ml, *F*_(1, 118)_ = 3.7, *p* = 0.05) and cortisone (aβ –0.1 (0.03) ng/ml, F_(1, 125)_ = 6.2, *p* = 0.01).

Median scores on the EPDS were 7 (0–19) at day 10 and 5 (0–25) at 4m CA, with approximately 29% of mothers scoring above the threshold indicative of postnatal depression (scores ≥10) at day 10 and 22% at 4m CA (Supplementary Table 1). GC concentration in HM was not different between mothers who did and who did not have postnatal depression symptoms at day 10 (Supplementary Table 1) and there was no correlation between day 10 total EPDS score and GC concentration in HM (Figure 4D). There was a weak negative correlation between total EPDS score at 4m CA and GC concentration in HM, which was statistically significant for cortisone (*r* = –0.1, *p* = 0.01), but not cortisol (*r* = –0.1, *p* = 0.2; Figure 4E). In adjusted model, total EPDS score at 4m CA was negatively associated only with cortisone in preterm HM (aβ –0.1 (0.04) ng/ml, *F*_(1, 131)_ = 5.7, *p* = 0.02).

### Maternal weight, height and body mass index

At 4m CA, maternal weight exhibited a weak negative correlation with GC concentration in HM, which was statistically significant for cortisone (*r* = –0.1, *p* = 0.03) but not cortisol (*r* = –0.1, *p* = 0.09). Maternal BMI also exhibited a weak negative correlation with GC concentrations in HM (cortisol: *r* = –0.1, *p* = 0.03; cortisone *r* = –0.2, *p* < 0.01). After model adjustment, maternal BMI was significantly associated only with cortisone concentrations (*F*_(2, 134)_ = 3.1, *p* = 0.05). Compared to mothers with healthy BMI, obese mothers produced HM with significantly lower cortisone concentrations (MD –1.0 (0.4) ng/ml, *p* = 0.04; Figure 4A).

### Feeding practices

At hospital discharge, almost 80% mothers were feeding their infant with only HM (either expressed HM or breastfeeding; Table 2). There was no association between GC concentrations in preterm HM and feeding practices at discharge and at 4m CA (data not shown).

### Infant factors

**FIGURE 5:**
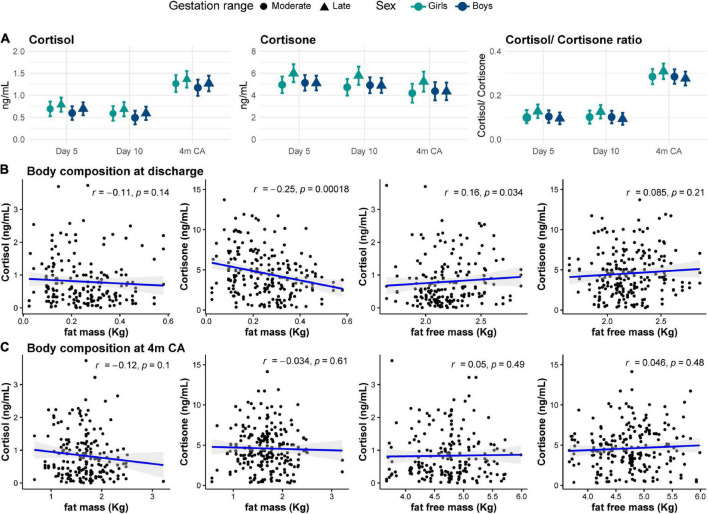
**(A)** Cortisol and cortisone concentrations in human milk for moderate (circles) and late (triangles) preterm girls (green) and boys (blue) at each collection age. Data are mean concentration (ng/ml) and standard error. Correlation between human milk cortisol and cortisone concentrations and body composition **(B)** at hospital discharge and **(C)** at 4 months corrected age (4m CA). Blue line and shaded area indicate regression line and 95% confidence intervals.

Both cortisol and cortisone concentrations in HM showed weak positive correlations with gestational age at birth (both *r* = 0.1, *p* < 0.05). Moderately preterm infants (32^+0^ to 33^+6^ weeks’ gestation) tended to receive HM with lower cortisone concentration than late preterm infants (34^+0^ to 35^+6^ weeks’ gestation; *cortisol* MD –0.1 (0.1) ng/ml, *p* = 0.07; *cortisone*: MD –0.4 (0.4) ng/ml, *p* = 0.03); however, these associations were no longer statistically significant after model adjustment (both adjusted *p* = 0.2; Supplementary Table 2). There were no differences in GC concentrations in preterm HM for girls and boys (Figure 5A).

### Size at birth

GC concentrations in HM were not significantly correlated with weight and length at birth. In adjusted model, cortisol was negatively associated only with birth head circumference (HC) *z*-score (aβ –0.01 (0.04) ng/ml, *F*_(1,173)_ = 4.2, *p* = 0.03; Supplementary Table 2).

### Growth

Changes in weight and HC *z*-scores between birth and discharge showed weak negative correlation with cortisone concentrations in HM (*r* = –0.2, *p* < 0.001; and *r* = –0.1, *p* = 0.049, respectively, data not shown). Growth between hospital discharge and 4m CA was not correlated with cortisol concentration in HM (all *r* < 0.4, *p* > 0.1, data not shown). There were no associations between growth and GC concentrations in preterm HM (Supplementary Table 2).

### Infant body composition

Body composition was assessed in a subset of participants at hospital discharge (*n* = 101) and at 4m CA (*n* = 102). Mean (SD) fat and fat free mass percentage at discharge were 10 (4)% and 90 (4)%, respectively, and at 4m CA were 25 (5)% and 75 (5)%, respectively (Table 2). At hospital discharge, absolute fat free mass showed a weak positive correlation with cortisol concentrations in HM (*r* = 0.2, *p* = 0.03), while fat mass showed a weak negative correlation with cortisone concentrations (*r* = –0.25, *p* < 0.001; Figure 5B). Body composition at 4m CA was not correlated with GC concentration in HM (all *r* < 0.2, *p* > 0.4; Figure 5C). There were no associations between body composition at either age and GCs in preterm HM (Supplementary Table 2).

## Discussion

In this longitudinal cohort of moderate to late preterm infants, we demonstrate that ANS administration is associated with lower concentrations of GCs in HM for at least 4 months, particularly following vaginal birth. ANS are widely used for prevention of neonatal morbidity among mothers at risk of preterm birth (10), but a significant proportion of women who receive antenatal glucocorticoids then go on to birth at, or near, term (39, 40). However, the potential effect of ANS on lactogenesis are seldomly investigated. We also confirm that cortisone is the predominant GC in preterm HM through to 4m CA, indicating that the mammary gland may inactivate cortisol into cortisone. Our findings indicate that concentrations of GCs in preterm HM are associated with many maternal and infant characteristics, including maternal obesity, postnatal stress and depression scores, infant gestational age, head circumference at birth and body composition at hospital discharge.

The development of the mammary gland is guided by “hormonal switches” during pregnancy and the postnatal period and both the order and timing of each hormonal exposure are essential for normal mammary development and lactation (12, 41). Mammary GC receptors increase during pregnancy and peak around the time of birth, remaining high during lactation until involution of the mammary gland at weaning (12), highlighting the essential role of GCs for mammary development (12, 42). During Lactogenesis I (or secretory differentiation), GCs act in synergy with prolactin and insulin in the mammary epithelial cells to induce differentiation of the lobule-alveolar system, development of rough endoplasmic reticulum and tight junctions, and expression of enzymes and milk protein genes, all of which are mechanisms essential for successful lactation (12). GCs also enable the lactogenic effect of prolactin by inducing the expression of prolactin receptors in the mammary gland (12). The second stage of lactogenesis (Lactogenesis II or secretory activation) occurs up to 4 days after birth, following the decline in progesterone levels, marked by copious milk production (43).

The concentration of cortisone in HM was up to 8 times higher than that of cortisol, consistent with previous studies (13, 15, 16, 24). The cortisol-to-cortisone ratio, usually taken to reflect 11β-HSD2 activity (44), was low throughout lactation. Thus, it seems likely that 11β-HSD2 may be active in the mammary gland to prevent inappropriately high maternal cortisol concentrations transferring into HM, thereby protecting the infant from exposure to excess glucocorticoid. An alternative possibility is that transferal of maternal cortisone into HM is greater than that for cortisol. The role of 11β-HSD2 in protecting the renal mineralocorticoid receptor from activation by cortisol and its role in the placenta in protecting the fetus from exposure to high levels of maternal cortisol have been well-described (44–47). Excessive maternal cortisol concentration during pregnancy due to maternal stress is linked to lower birth weight (48, 49), shorter gestation (49) and impaired neonatal stress regulation (50). Reduced placental 11β-HSD2 activity, measured in cord blood at birth, has been associated with high systolic blood pressure at 3 years of age (51). Exposure to antenatal synthetic GCs, which cross the placental barrier without inactivation by 11β-HSD2, may lead to insulin resistance in adulthood (52). 11β-HSD2 localization in other mineralocorticoid-responsive tissues, including salivary and sweat glands, epithelial tissue (44), and in ductal epithelial mammary cells of non-lactating females (53), also has been reported. Nevertheless, more studies are needed to elucidate the expression and activity of mammary 11β-HSD2 during lactation.

Cortisol and cortisone were highly correlated, with strength of correlation decreasing throughout lactation possibly due to reduction in sample size at 4 months CA. The concentrations of cortisol detected in our study are lower than reported in previous studies (24, 27, 28) including one on preterm HM (21). Possible reasons include high exposure to ANS in our cohort, and differences in study population or methodology, since most studies were conducted in Caucasian (15, 21, 27, 28) or European (13, 16, 24, 26) mothers, included mothers of full term infants (15, 16, 22, 24), employed cross-sectional analysis of mature HM (15, 24), and used different analytical methods (27). The concentrations we report are, however, similar to one study involving extremely and very preterm infants (13). We previously have demonstrated that medium chain fatty acids and metabolic hormones in preterm HM differed by maternal ethnicity (54, 55) but it is unclear if ethnicity influences the concentration of GCs in HM. Despite the multi-ethnic population, our study is not fully representative of the general population of woman giving birth in New Zealand (56), with Māori and Caucasian mothers being under-represented and Asian and Pacific mothers over-represented compared to the national birthing population.

Administration of ANS is widely recommended for woman at risk of preterm birth to reduce neonatal respiratory morbidity and mortality risks (10) and the majority of mothers in our study received some ANS. Our findings suggest that receipt of even a single dose of ANS given less than 24 h before birth is associated with lower GCs concentration in HM through to 4 months, even among mothers who birth vaginally. Possible explanations include suppression of the maternal HPA axis in response to the high levels of exogenous glucocorticoid (57), disruption of the hormonal switches that lead to secretory activation (12, 41) or other underlying health complication leading to ANS administration due to risk of preterm birth, such as chorioamnionitis or premature rupture of membranes. It is unlikely that maternal HPA axis suppression following administration of ANS persists for 4 months postpartum, implying a more profound effect of ANS on mammary gland development and lactation, although we do not have maternal plasma cortisol concentrations with which to compare the HM concentrations.

Along with the rise in maternal GCs levels in late gestation (7), studies in both rats and cattle demonstrate that GC receptors in the mammary gland increase during pregnancy, peak around parturition and return to pregnancy levels during lactation (12, 42). Studies in lactating rats suggest that administration of synthetic GCs (dexamethasone 21-acetate) down-regulates GC receptors in mammary gland cytosol, with receptor binding affinity remaining low even 48 h after synthetic GC withdrawal (58). In humans, Henderson et al. reported reduced milk volume among mothers of preterm infants who received antenatal steroids 3–9 days before birth compared with mothers giving birth up to 2 days after ANS treatment, but markers of secretory activation such as lactose and citrate did not differ between study groups (59). Since timing of ANS administration often coincides with Lactogenesis I, it is possible that down-regulation of mammary GC receptors leads to low GC concentration in HM; however, the length of suppression observed in our study is surprising and we are unaware of other human studies investigating the long-term effects of ANS on lactogenesis.

Altered HPA axis activity has been associated with several pathological conditions, including hypertension, dyslipidemia, and obesity (6). On one hand, acute stress often induces short-term rises in plasma cortisol concentrations (6, 9). In contrast, chronic stress and prolonged exposure to GCs may induce structural changes to the HPA axis, leading to suppression of HPA activity and blunted cortisol reactivity, often referred to as “wear and tear” effect or allostasis (60), as reported in chronic depression and anxiety (61) and obesity (62). Our finding that maternal obesity is associated with lower GC concentrations in HM is consistent with data from a large Finnish cohort (24). The association between higher scores in both stress and postnatal depression and lower GCs in HM is less clear and contrary to the general assumption of positive relationship between stress and cortisol. Psychosocial distress at 3 months postpartum as a composite of maternal stress, anxiety and postpartum depression symptoms has been reported to be positively associated with cortisol concentrations in HM (22). Whereas Lindberg et al. identified a negative correlation between HM cortisol concentrations and maternal anxiety, but not postpartum depression symptoms, 3 months after birth (26), the large Finnish cohort found no association between maternal distress and GCs in HM at 3 months postpartum (24). Since both PSS and EPDS are indicative of mid- to long-term stress and depressive symptoms, they likely reflect chronic rather than acute stress and this may explain the association with lower maternal GC concentrations. Of note, none of these studies focused on mothers of preterm infants who often experience stressful situations in the postnatal period.

We did not find significant associations between GC concentrations in HM and size at birth, infant growth or body composition, apart from weak correlations between cortisone and birth head circumference *z*-score, cortisol and infant fat free mass, and cortisone and fat mass. One study previously has shown that higher HM cortisol concentration at 3 months was predictive of lower body mass index percentile during first 2 years of life (27). However, others reported positive associations between HM cortisol concentrations and infant fat mass in the first year (28). Therefore, the effect of HM GCs on postnatal growth and body composition remains inconclusive.

Studies on longitudinal changes of GC concentrations in HM have yield conflicting results. While some have reported that these remain unchanged over time (21, 28), others suggest an increase in cortisol concentrations as lactation progresses (22). It is possible that lower GC concentrations that we observed in early lactation may be due to reduced maternal HPA axis activity following birth and cessation of placental corticotrophin-releasing hormone (CRH) secretion (7), since both of our early lactation samples fell within first 10 postnatal days. Effects of lactation stage on HM composition, including of hormones such as GCs, may have important implications for HM donation and nutrition of preterm infants. GCs are preserved during pasteurisation (63) and donor HM may have different profile of hormones when compared to mothers’ own milk, especially when circadian cycles are not considered (5, 25). Thus, a hormonal mis-match may occur when infants are not breastfeeding or not receiving HM expressed at similar times to feeding, with unclear implications for infant development and metabolic programming.

### Strengths and limitations

Our study has several strengths, including the large cohort of moderate to late preterm infants and standardized HM collection protocol, which ensured that majority of samples obtained were collected from full breast expression in the morning. The superiority of LC-MS for quantification of HM GCs (64) and the standardized growth and body composition assessments permitted robust investigation of associations between HM GCs and postnatal growth. Although detailed nutritional intake data were collected as part of the DIAMOND trial, an estimation of GC intake was not possible since most moderate and late preterm infants are also breastfed during their hospital stay and HM samples were limited to a morning sample on postnatal day 5 and day 10, which would not accurately reflect the diurnal GC variation in HM and the actual intake by the infant. Further, as this study is a secondary cohort analysis, with sample size determined by the participants of the main trial who provided HM samples, it is possible that unknown factors not investigated (such as pre-pregnancy BMI or maternal body composition) could have confounded the associations identified, and thus caution is warranted when extrapolating current findings to another population.

### Future directions

Further research is needed to elucidate the effect of ANS on lactogenesis and potential impacts on the preterm infant. Given that preterm infants often receive HM at times mis-matched to collection times, the potential short- and long-term effects of feeding preterm infants HM with different hormone levels (hormonal mis-match), such as HM expressed at different time of the day or donor HM (often mature HM pooled from many donors) requires further research. Assessment of circulating GC concentrations in both infant and mother using saliva and/or plasma samples in parallel with HM collection will help to investigate further the mother-milk-infant communication *via* HM hormones. Furthermore, more animal and *in vitro* studies are required to characterize the expression and activity of mammary 11β-HSD2 during lactation.

## Conclusion

The mammary gland appears to protect the infant from cortisol through inactivation into cortisone. GCs in HM were inversely correlated with maternal stress, postnatal depression and BMI, and very weakly correlated with infant size at birth, in-hospital growth, and body composition at hospital discharge. ANS may have lasting effects on the concentration of GCs in HM and, given that most mothers at risk of preterm labor are exposed to ANS, more research is needed to understand the implications of ANS on lactogenesis and whether HM GCs can influence HPA axis function, growth and development of moderate and late preterm infants.

## Data availability statement

The raw de-identified individual participant data supporting the conclusions of this article will be made available by the authors (including data dictionaries) upon reasonable request and after review by the Data Access Committee of the Liggins Institute. Proposals should be submitted to the corresponding author.

## Ethics statement

The studies involving human participants were reviewed and approved by New Zealand Health and Disability Ethics Committee (number: 16/NTA/90). Written informed consent to participate in this study was provided by the participants’ legal guardian/next of kin.

## Author contributions

MM assisted with sample collection, carried out statistical analysis, interpreted the results, and drafted the manuscript. TA designed the randomized controlled trial, provided funding for sample and data collection, and contributed to the manuscript development. MV contributed to interpretation of results and the manuscript development. JH designed the randomized controlled trial and contributed to manuscript development. LG designed breastmilk collection protocol and contributed to the manuscript development. FB designed the randomized controlled trial, provided funding for sample and data collection, contributed to interpretation of results, and the manuscript development. All authors contributed to the manuscript and approved the submitted version.
