# Supplementary material for: Glucocorticoids in preterm human milk
Source: Front Nutr. 2022 Sep 27;9:965654. doi: 10.3389/fnut.2022.965654 (PMC9552215; doi:10.3389/fnut.2022.965654)
Supplement: Supplementary file 1 [file Data_Sheet_1.pdf]

Glucocorticoids in preterm human milk

Supplementary material

Muelbert Mariana[1], Tanith Alexander[1,2], Mark H. Vickers[1], Jane E. Harding [1], Laura Galante[1], Frank H. Bloomfield[1*] for the DIAMOND study group

[1]Liggins Institute, University of Auckland, New Zealand

[2]Neonatal Unit, Kidz First, Middlemore Hospital, Auckland, New Zealand

*** Correspondence:**
Frank H. Bloomfield
f.bloomfield@auckland.ac.nz

Supplementary tables

Supplementary Table 1 – Association between concentration of GCs in preterm HM and maternal characteristics.

		Cortisol		*Cortisone*		*Cortisol-to-cortisone ratio*	
	N (%)	*Mean (SE)*	*aMean (SE)*	*Mean (SE)*	*aMean (SE)*	*Mean (SE)*	*aMean (SE)*
**Pregnancy characteristics**[††]	**n=170**						
Diabetes in pregnancy							
No	135 (79)	0.6 (0.4)	0.8 (0.04)	5.0 (0.3)	4.9 (0.3)	0.1 (0.01)	0.1 (0.01)
Yes	35 (21)	0.6 (0.1)	0.8 (0.1)	4.5 (0.2)	4.9 (0.2)	0.1 (0.01)	0.2 (0.01)
**Maternal characteristics**[††]	**n=170**						
Maternal age							
≤30 years	68 (40)	0.6 (0.1)	0.8 (0.1)	4.6 (0.3)	4.8 (0.3)	0.1 (0.01)	0.2 (0.01)
>30 years	102 (60)	0.6 (0.04)	0.8 (0.04)	4.7 (0.2)	5.0 (0.2)	0.1 (0.01)	0.1 (0.01)
Ethnicity							
Caucasian	66 (39)	0.7 (0.1)	0.8 (0.1)	5.0 (0.3)	5.3 (0.3)	0.1 (0.01)	0.2 (0.01)
Asian	54 (32)	0.6 (0.1)	0.8 (0.1)	4.3 (0.3)	4.6 (0.3)	0.1 (0.01)	0.2 (0.01)
Māori	20 (12)	0.5 (0.1)	0.6 (0.1)	4.9 (0.5)	4.7 (0.5)	0.1 (0.02)	0.1 (0.02)
Pasifika	27 (15)	0.6 (0.1)	0.7 (0.1)	4.3 (0.4)	4.5 (0.4)	0.1 (0.05)	0.2 (0.01)
Other	3 (2)	1.0 (0.3)	1.1 (0.2)	5.6 (1.2)	5.7 (1.2)	0.2 (0.05)	0.2 (0.04)
New Zealand Deprivation Index							
Q1 (1,2)	27 (16)	0.6 (0.1)	0.8 (0.1)	5.2 (0.4)	5.7 (0.4)	0.1 (0.06)	0.1 (0.01)
Q2 (3,4)	39 (23)	0.7 (0.1)	0.9 (0.1)	4.9 (0.3)	5.2 (0.3)	0.2 (0.06)	0.2 (0.01)
Q3 (5,6)	24 (14)	0.5 (0.1)	0.6 (0.1)	4.3 (0.5)	4.4 (0.4)	0.1 (0.06)	0.1 (0.02)
Q4 (7,8)	30 (18)	0.7 (0.1)	0.8 (0.1)	4.3 (0.4)	4.5 (0.4)	0.2 (0.06)	0.2 (0.01)
Q5 (9,10)	40 (29)	0.6 (0.1)	0.8 (0.1)	4.6 (0.3)	4.9 (0.3)	0.2 (0.06)	0.2 (0.01)
Education level							
Secondary education or lower	51 (30)	0.6 (0.1)	0.8 (0.1)	4.5 (0.3)	4.7 (0.3)	0.1 (0.01)	0.2 (0.01)
Post-secondary education	30 (18)	0.7 (0.1)	0.8 (0.1)	4.7 (0.4)	4.9 (0.4)	0.1 (0.01)	0.2 (0.01)
Tertiary education (≥university)	89 (52)	0.6 (0.05)	0.8 (0.05)	4.7 (0.2)	5.0 (0.2)	0.1 (0.01)	0.1 (0.01)
**Perceived stress[1] and depression[2]**							

		Cortisol		Cortisone		Cortisol-to-cortisone ratio	
	N (%)	*Mean (SE)*	*aMean (SE)*	*Mean (SE)*	*aMean (SE)*	*Mean (SE)*	*aMean (SE)*
Perceived stress (~day 10) ††	n=154						
Low	56 (36)	0.6 (0.1)	0.7 (0.1)	4.9 (0.3)	5.0 (0.3)	0.1 (0.01)	0.1 (0.01)
Moderate	91 (59)	0.6 (0.05)	0.8 (0.05)	4.4 (0.2)	4.8 (0.2)	0.1 (0.01)	0.2 (0.01)
High	7 (5)	0.4 (0.2)	0.7 (0.2)	4.2 (0.8)	4.8 (0.8)	0.1 (0.03)	0.1 (0.03)
Perceived stress (follow-up) ††	n=147						
Low	75 (51)	0.7 (0.6)	0.8 (0.1)	5.0 (0.2)	5.2 (0.3)	0.1 (0.05)	0.1 (0.01)
Moderate	69 (47)	0.6 (0.1)	0.8 (0.1)	4.3 (0.3)	4.5 (0.3)	0.1 (0.01)	0.2 (0.01)
High	3 (2)	0.5 (0.3)	0.6 (0.2)	4.6 (1.2)	5.0 (1.1)	0.1 (0.1)	0.1 (0.04)
Postnatal depression (~day 10) ††	n=153						
No	105 (69)	0.6 (0.04)	0.8 (0.05)	4.6 (0.2)	5.0 (0.2)	0.1 (0.01)	0.2 (0.01)
Yes	48 (31)	0.6 (0.1)	0.8 (0.07)	4.4 (0.3)	4.7 (0.3)	0.1 (0.01)	0.1 (0.01)
Postnatal depression (follow-up) ††	n=148						
No	116 (78)	0.7 (0.05)	1.1 (0.1)	4.8 (0.2)	4.9 (0.2)	0.1 (0.01)	0.1 (0.01)
Yes	32 (22)	0.6 (0.1)	1.2 (0.2)	4.3 (0.4)	4.5 (0.4)	0.1 (0.01)	0.2 (0.01)

Linear regression model mean estimates (ng/mL) and standard error (SE). Different superscript letters indicate groups significantly different. †adjusted for antenatal steroids course; § adjusted for lactation stage; ††adjusted for lactation stage and antenatal steroids course. aMean: adjusted mean estimate; PN: postnatal age; Q: Quintiles of social deprivation; HM: human milk; BMI: body mass index. *p<0.05; **p<0.01; ***p<0.001.

[1]Perceived stress: Perceived Stress Scores (PSS) ranging from 0-13, 14-26 and 25-40 points were considered low, moderate and high perceived stress, respectively (Cohen et al., 1983).

[2]Perceived postnatal depression: Edinburgh Postnatal Depression Scale (EPDS) scores equal to 10 or above were considered indicative of postnatal depression (Cox et al., 1987; Wisner et al., 2002).

Supplementary Table 2– Association between glucocorticoid concentrations in preterm HM and infant characteristics.

		Cortisol		*Cortisone*		*Cortisol-to-cortisone ratio*	
	N (%)	***Mean (SE)***	***aMean (SE)***	***Mean (SE)***	***aMean (SE)***	***Mean (SE)***	***aMean (SE)***
**Birth size for gestational age**							
SGA	24 (12)	0.8 (0.1)	0.9 (0.1)	4.4 (0.4)	4.8 (0.5)	0.1 (0.02)	0.2 (0.02)
AGA	160 (84)	0.7 (0.04)	0.8 (0.05)	4.8 (0.2)	5.0 (0.2)	0.1 (0.01)	0.2 (0.01)
LGA	7 (4)	0.7 (0.2)	0.7 (0.2)	4.5 (0.8)	4.6 (0.8)	0.1 (0.03)	0.2 (0.03)
	Median (range)	***β (SE)***	***aβ (SE)***	***β (SE)***	***aβ (SE)***	***β (SE)***	***aβ (SE)***
**Gestational age (weeks)**	33 (32-35)	0.1 (0.04)	0.04 (0.04)	0.3 (0.2)*	0.1 (0.2)	0.003 (0.01)	0.001 (0.01)
**Birth anthropometrics**							
Weight (z-score)	-0.05 (-2.6, 2.5)	-0.03 (0.04)	-0.1 (0.04)	0.2 (0.2)	0.03 (0.4)	-0.001 (0.01)	-0.01 (0.01)
Length (z-score)	0.3 (-3.8, 3.2)	-0.01 (0.04)	-0.03 (0.04)	0.1 (0.1)	0.1 (0.1)	0.001 (0.01)	-0.001 (0.01)
HC (z-score)	0.4 (-2.0, 2.6)	-0.05 (0.04)	-0.1 (0.04)*	0.2 (0.2)	0.04 (0.2)	-0.005 (0.01)	-0.01 (0.01)
**In-hospital growth**							
Growth velocity (g/Kg.day)	7.7 (-19.3, 19.0)	-0.03 (0.1)	-0.004 (0.2)	-0.02 (0.04)	-0.02 (0.04)	-0.01 (0.7)	0.1 (1.0)
Weight z-score change	-0.8 (-1.8, 0.5)	-0.001 (0.1)	0.0003 (0.01)	-0.001 (0.003)	-0.001 (0.003)	0.002 (0.1)	0.01 (0.1)
Length z-score change	-0.4 (-2.5, 3.1)	0.01 (0.03)	0.01 (0.04)	0.002 (0.01)	0.002 (0.01)	0.02 (0.2)	0.02 (0.2)
HC z-score change	-0.4 (-2.2, 1.4)	0.004 (0.03)	0.02 (0.03)	-0.004 (0.01)	-0.002 (0.01)	-0.01 (0.2)	0.05 (0.2)
**Birth to follow-up**							
Weight z-score change	-0.03 (-2.8, 2.1)	-0.006 (0.04)	0.005 (0.04)	0.1 (0.3)	-0.0001 (0.01)	-0.05 (0.2)	0.005 (0.2)
Length z-score change	0.3 (-2.8, 4.8)	0.005 (0.05)	0.02 (0.06)	-0.002 (0.01)	-0.002 (0.01)	-0.06 (0.3)	-0.02 (0.3)
HC z-score change	0.3 (-2.1, 2.4)	0.0001 (0.003)	0.005 (0.03)	0.001 (0.01)	0.001 (0.01)	-0.03 (0.1)	-0.02 (0.2)
**Body composition (discharge)**							
Fat free mass (Kg)	2.2 (1.6, 2.9)	0.004 (0.01)	0.001 (0.01)	0.002 (0.004)	0.001 (0.003)	0.02 (0.06)	0.02 (0.1)
Fat mass (Kg)	0.2 (0.02, 0.6)	-0.0004 (0.003)	-0.0005 (0.003)	-0.0003 (0.001)	-0.0003 (0.001)	0.002 (0.01)	0.002 (0.02)
Fat mass index (Kg/m^2)	1.2 (0.1, 2.3)	-0.001 (0.01)	-0.002 (0.01)	-0.001 (0.002)	-0.001 (0.003)	0.01 (0.05)	0.005 (0.06)
Fat free mass index (Kg/m^2)	9.8 (8.0, 11.0)	0.01 (0.03)	0.01 (0.03)	0.01 (0.01)	0.004 (0.01)	0.01 (0.1)	0.004 (0.2)
**Body composition (follow-up)**							
Fat free mass (Kg)	4.8 (3.7, 6.0)	-0.002 (0.02)	-0.003 (0.02)	-0.0001 (0.001)	-0.0005 (0.005)	-0.01 (0.1)	-0.001 (0.1)
Fat mass (Kg)	1.7 (0.6, 3.2)	-0.001 (0.01)	-0.003 (0.01)	-0.0001 (0.002)	-0.0001 (0.002)	-0.005 (0.04)	-0.01 (0.05)

Fat mass index (Kg/m^2)	4.1 (1.5, 7.3)	-0.003 (0.02)	-0.005 (0.02)	-0.0004 (0.005)	-0.000001 (0.005)	-0.01 (0.1)	-0.02 (0.1)
Fat free mass index (Kg/m^2)	11.9 (9.1, 14.0)	-0.005 (0.03)	-0.001 (0.04)	0.0005 (0.01)	0.0003 (0.01)	-0.02 (0.1)	0.02 (0.2)

Linear regression model mean estimates or ß coefficient (ng/mL) and standard error (SE). Model adjusted for sex, antenatal steroid course and lactation stage. aMean: adjusted mean estimate; AGA: appropriate for gestational age; LGA: large for gestational age; SGA: small for gestational age; HC: head circumference. *$p<0.05$;
